# Influence of Foehn on Aortic Aneurysm Ruptures in Southern Germany

**DOI:** 10.3390/jcm14093104

**Published:** 2025-04-30

**Authors:** Elena Streck, Irena Kaspar-Ott, Oksana Radu, Stefan Schiele, Hans-Henning Eckstein, Gernot Müller, Elke Hertig, Alexander Hyhlik-Dürr

**Affiliations:** 1 Vascular Surgery, Faculty of Medicine, University of Augsburg, Stenglinstraße 2, 86156 Augsburg, Germany; 2 Regional Climate Change and Health, Medical Faculty, University of Augsburg, Universitätsstraße 2, 86159 Augsburg, Germany; 3 Department for Vascular Surgery, Klinikum Rechts der Isar, Technical University Munich, Ismaningerstraße 22, 81675 München, Germany; 4 Institute of Mathematics, University of Augsburg, Universitätsstraße 14, 86159 Augsburg, Germany

**Keywords:** aortic aneurysm rupture, acute aortic syndrome, foehn wind, weather

## Abstract

**Background/Objectives:** Foehn, a warm, dry wind blowing down into the valleys of a mountain, is a typical weather condition in southern Germany. Until now, there have been no data regarding the impact of foehn on aortic aneurysm ruptures in the Alpine regions, analyzed in this study. **Methods:** In this retrospective German dual-center study (University Augsburg, University Munich) were enrolled 152 patients with a rupture of the thoracic aortic aneurysm (rTAA), abdominal aortic aneurysm (rAAA), and thoracoabdominal aortic aneurysm (rTAAA), living within 20 km of weather measuring stations. We analyzed the risk factors for aortic aneurysm rupture dependent on weather changes in southern Germany using the meteorological data from January 2010 to December 2019. **Results:** The most common form of ruptured aortic aneurysm (rAA) was abdominal aortic rupture in both sexes (64.5% men, 17.1% women). The incidence rate of rAAA from Augsburg and Munich was 20.4% in spring, 26.3% in autumn, 28.9% in summer, and 24.3% in winter. Indeed, in Augsburg, rAAAs occurred most often during winter months (32%), while in Munich the majority of cases occurred during summer (32%). We observed that aortic ruptures on days with a tendency for southerly wind flow and lower air pressure were correlated with foehn in southern Germany. **Conclusion:** The occurrence of foehn days could be a relevant risk factor for increased incidence of rAA.

## 1. Introduction

An aortic aneurysm, known as a “silent killer”, can cause a rupture in any section of the dilated or weakened aortic wall: the thorax (TAA: thoracic aortic aneurysm), the abdomen (AAA: abdominal aortic aneurysm), or combination of both (TAAA: thoracoabdominal aortic aneurysm) [1]. Ruptured aortic aneurysm (rAA) is a life-threatening acute aortic syndrome (AAS). In 2010, ruptured aortic aneurysms ranked as the 13th leading cause of death in Germany [2]. Without immediate treatment, approximately 43–46% of patients die prior to hospital admission or intervention, and 26–32% of patients die after surgical therapies [3].

The most commonly known risk factors associated with aortic aneurysm rupture include aneurysm diameter, patient age older than 65 years, uncontrolled hypertension, current smoking, and Caucasian ethnicity [4]. The available literature suggests that the rupture risk in women is four times higher at a diameter 5.0–5.9 cm than in males, possibly due to differences in the biomechanical properties of the aneurysm wall [5]. Aortic aneurysm in females usually present late and grow more rapidly than males [6]. Further risk factors are dependent on wall stress, aneurysm shape and geometry, intraluminal thrombus, wall thickness, calcification, and metabolic activity, which may influence the rupture risk [7].

Some studies suggest that weather conditions such as low air pressure and low air temperature may have an influence on the rupture of an aortic aneurysm [8,9,10]. The results are not uniform and cannot be confirmed from numerous studies, probably due to seasonal changes or meteorological conditions in different countries [11]. In 2019, the meta-analysis study from Choong et al. showed that autumn and winter are significantly associated with a higher incidence of ruptures of abdominal aortic aneurysms (rAAAs). The highest incidence of rAAAs was in December, and the lowest in July. Results about the relation of sunlight hours to rAAA are not comparable [12,13]. Kozka et al. reported no significant association between moon phases and risk of rAAA [14].

In 2022, our group published ten years of meteorological data regarding the influence of weather on the occurrence of rAAs in southern Germany (data from Augsburg and Munich) and established a new method to determine the influence of meteorology on ruptured aortic aneurysms [15]. We demonstrated that specific weather conditions, with south to southwest air flow, which can be attributed to higher air pressure south of the Alps and lower pressure in northern Europe, correlated with foehn. There is a dearth of research regarding foehn’s impact on aortic aneurysm ruptures. This is the first study investigating the effect of foehn among patients with aortic aneurysm rupture located in a foehn area in southern Germany. The next aim of this study was to evaluate whether or not a possible relationship between weather types (WTs) and rAA exists.

## 2. Materials and Methods

### 2.1. Patients

Between January 2010 and December 2019, 248 patients with aortic aneurysm rupture in different parts of the aorta (thorax, abdomen, and combination of both) were retrospectively identified using International Classification of Diseases codes and the inclusion keywords “aortic aneurysm rupture” from a database at the Augsburg University Hospital and University Hospital of the Technical University Munich (TUM).

Baseline information on age, gender, and cardiovascular risk factors are shown in Figures S1–S3. The meteorological data on all patients with rupture of their aortic aneurysm were analyzed using season and month distribution over ten years.

### 2.2. Aneurysm Measurements

Aneurysm size at the date of rupture was measured as the maximum diameter of the aneurysm on computed tomography angiography (CTA) scan (Siemens, Forchheim, Germany).

### 2.3. Meteorological Data

Daily weather data for air temperature (minimum: tmin; maximum: tmax; and daily mean: tmean), atmospheric pressure (slp), relative humidity (rhum), precipitation (prec), wind speed (windv) and wind direction (windd) for the years 2010–2019 were provided by the German Weather Service (DWD) at the DWD stations Augsburg-Mühlhausen and Munich-City (DWD Climate Data Center (CDC) 2021). We used daily mean values of ERA5 reanalysis for atmospheric pressure (slp) for 2010–2019 [15]. The ERA5 data covers the area from 25° N–70° N and 25° W–40° E, with a 1 × 1° resolution.

### 2.4. Jenkinson–Collison (JC) Weather Types

To investigate a possible relationship between weather types (WTs) and rAA, daily Jenkinson–Collison (JC) weather types were calculated using air pressure data from the ERA5 reanalysis, with Augsburg and Munich as the centers of the spatial domain [16]. The JC method was created to objectively classify the 27 weather types in the Lamb weather type system. These include eight purely advective (directional) types determined by wind direction (e.g., SW for southwest); types A and C, which represent anticyclonic and cyclonic pressure patterns, respectively, with no clear flow direction; 16 hybrid types, which combine directional types with either anticyclonic isobaric curvature (e.g., AN for anticyclonic northerly) or cyclonic curvature (e.g., CSE for cyclonic southeasterly); and type U (indifferent), which describes patterns with weak pressure gradients where neither flow direction nor vorticity is identifiable.

### 2.5. Definition of Foehn-like Events

Foehn is a type of dry, warm, downslope wind that occurs on the leeward side of mountains. Typically, foehn weather conditions occur about two to eight times a month in spring and autumn but can also occur in winter [17]. It is challenging to determine whether a foehn event occurred in Augsburg or Munich on a given day purely from measured data and without expert knowledge. Nevertheless, it can be assumed that some meteorological conditions must prevail that allow a definition of at least foehn-like conditions. These are defined as follows within the measured data from the DWD: The wind direction should have values between 125° and 235° (SE–SW) at the measurement sites, the wind speed is higher than the annual mean, the relative humidity is lower than the mean humidity of the respective month, and the mean daily temperature is higher than the mean temperature of the respective month.

### 2.6. Statistical Analysis

All statistical analyses were performed using GraphPad Prism software (version 9.0.1) and R software (version 3.6.1). The comparison between groups was performed using the unpaired Student’s *t*-test. Variables were expressed as mean ± standard deviation. *p* values < 0.05 were considered statistically significant.

## 3. Results

### 3.1. Baseline Patient Characteristics

After narrowing down the patient numbers in relation to their place of residence to a radius of 20 km around the weather measuring stations, we identified 152 out 248 aortic aneurysm ruptures from the Augsburg University Hospital and University Hospital of the TUM. It was necessary to exclude 96 patients because weather is a local phenomenon, and therefore a limit of 20 km was set. Higher distances could cause a range of heterogenous effects on aortic aneurysm rupture, what could constitute a bias [18].

In the retrospective dual-center study, 114 men (66%) and 38 women (33%) were enrolled, resulting in a 3:1 ratio. The mean patient age of men was 76 ± 9.7 years. Women were significantly older at the time of rupture of their aortic aneurysm (81.3 ± 7.5 years, *p* = 0.003) (Supplemental Materials, Figure S1).

In the Supplemental Materials, Figure S2 shows the typical comorbidities in the cardiovascular risk patients. Most patients suffered from hypertension (55.9%), the best-known risk factor for rupture of aortic aneurysms. COPD, type II diabetes, and renal failure were in the range between 11% and 15%. A total of 152 ruptures of aortic aneurysm were subdivided into rTAA, rTAAA, and rAAA and correlated with gender. The distribution of ruptures was as follows: 7.9% were rTAA (3.9% men, 3.9% women), 10.5% rTAAA (6.6% men, 3.9% women), and 81.6% rAAA (64.5% men, 17.1% women) (Supplemental Materials, Figure S3).

The maximum aorta diameter of different morphologies of aortic aneurysms by rupture are shown in the Supplemental Materials, Figure S4. The mean of aortic diameter of the ruptured aortic aneurysm does not differ between different morphologies of aortic aneurysms.

The number of treated rAAs per year from 2010–2019 is shown in the Supplemental Materials, Figure S5. A total of 19 patients with rAA per year was the maximum number in both University Hospitals in Augsburg and Munich; the minimum number was 11 rAAs per year. The frequency of rAA from year to year shows no trend between the two cities studied.

### 3.2. Effect of Seasonal and Monthly Weather Changes on rAAs

**Figure 1:**
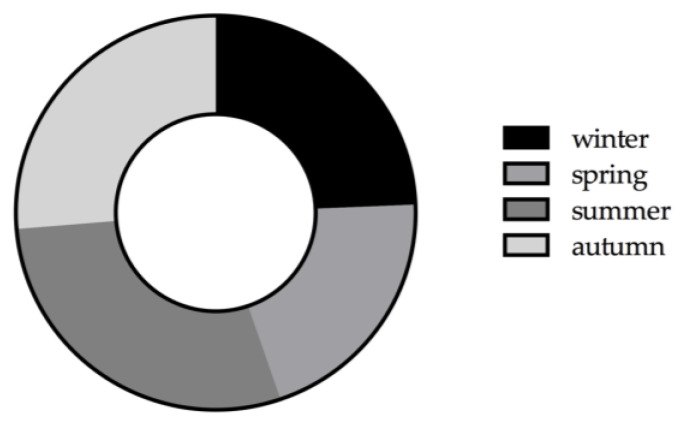
Seasonal distribution of 152 ruptured aortic aneurysms at the Augsburg University Hospital and University Hospital of the Technical University Munich between January 2010 and December 2019. Summer: 28.9% incidence rate; autumn: 26.3% incidence rate; winter: 24.3% incidence rate; spring: 20.4% incidence rate.

Figure 1 shows the seasonal distribution of 152 rAAs from Augsburg and Munich: spring (20.4%), autumn (26.3%), summer (28.9%), and winter (24.3%). In Augsburg, the most rAAs occurred in winter from 2010 to 2019, and the fewest in the months of March, May, and September. In Munich, the most cases occurred in summer, and the fewest in the months of January, February, and May. The detailed data on the seasonal distribution of each city were previously published by Kaspar-Ott et al. in 2022 [15].

We also analyzed the monthly distribution of 152 rAAs, including the subtypes of rAA (rTAA, rTAAA, and rAAA), over ten years in the Supplemental Materials, Figure S6.

The in-hospital mortality rate was higher in winter (42.6%), and the lowest was in spring (10.6%). The most rAA deaths (17%) were reported in December (Supplemental Materials, Figures S7 and S8). One possible reason is that the cold temperature contributes to increasing sympathetic nervous activity, affecting blood pressure [19]. In March and September, there was no mortality for patients with rAAs.

### 3.3. Results for the JC Weather Types—Exemplary for Winter

**Figure 2:**
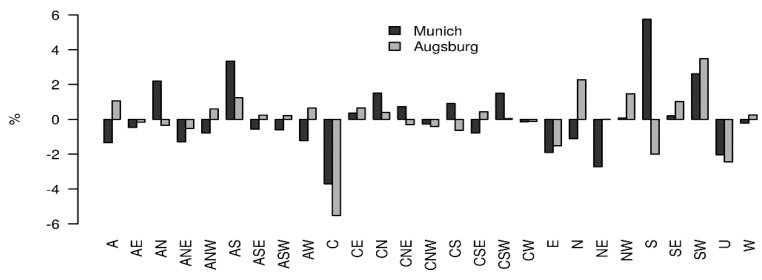
Percentage differences of Jenkinson–Collison circulation patterns in Augsburg and Munich between rAAs (plus a time lag of 7 days) and periods without rAAs in October–January 2010–2019. A = anticyclonic, C = cyclonic, S = South, W = West, N = North, E = East, U = indifferent weather type.

Figure 2 shows the differences in occurrence frequencies (in percent) of weather types (WT) in Augsburg and Munich between the days of rAA (including the seven preceding days) and the days on which no rAAs occurred in the winter months, from October to January. It is noticeable that at both locations, the weather types with southerly flow tendency are more frequently represented in the case of rAA. This mainly concerns the WTs AS (anticyclonic south) and SW (southwest). WTs with cyclonic flow direction, as well as northerly and easterly flow directions, on the other hand, are less frequently encountered before or on the day of the rAA. In Augsburg, as well as in Munich, this concerns the weather situations ANE, C, and E. The weather situation U (indifferent weather type), which is characterized by low-flow conditions, also occurs less frequently.

### 3.4. Association of Foehn-like Days and rAA

Applying the criteria defined above to the DWD datasets, an average of 302 events remain for Augsburg and Munich for the years 2010–2019 that are considered foehn-like. This corresponds to the frequency data of experts [20], if one assumes that, on average, five foehn events occur per month for the relevant seasons (six months), resulting in a total of 300 foehn events over the course of ten years. rAAs that occurred during a foehn-type event are therefore declared as foehn-rAA.

**Figure 3:**
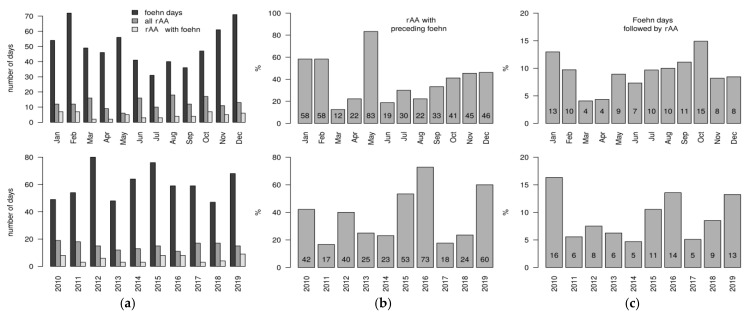
(**a**) Number of foehn-like days for Augsburg and Munich, number of rAAs, and number of rAAs that occurred during a foehn event or on the six following days (foehn–rAA). (**b**) The proportion of foehn–rAAs to all rAAs in %. (**c**) The probability of rAAs in % during or after a foehn event. The top row shows the monthly frequencies; the bottom row shows the frequencies for each year.

Figure 3a–c shows the distribution of foehn events in Augsburg and Munich on both a monthly and annual basis. More foehn-like events occur in the winter months than in summer, and the interannual variability is very high, with most foehn events in 2012, 2015, and 2019 (Figure 3a). The middle column in Figure 3b shows, in percentage terms, how many of all rAAs that occurred were rAAs preceded by a foehn event (foehn-rAA). Accordingly, in January and February, 58% of all rAAs that occurred followed a foehn-type weather event. Again, values are generally higher in the winter months than in the summer, except for May, when over 83% of rAAs were associated with a foehn event. There are large differences across the years. For example, in 2011 less than 17% of all rAAs were associated with foehn weather conditions, compared to nearly 73% in 2016.

The last column in Figure 3c indicates the frequency of rAAs preceded by foehn weather conditions. Foehn days occurring in October will be followed by rAAs in the following week, with a probability of 15%. The probabilities are above 10% in January, August, and September. The lowest probabilities, approximately 4%, are in March and April. Again, the interannual variability is relatively high.

### 3.5. Composites of Air Pressure Fields That Have an Influence on the Occurrence of rAA in Southern Germany: The ALPINE Foehn

**Figure 4:**
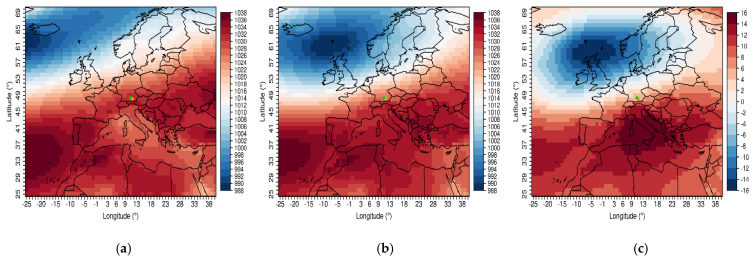
Composites of the air pressure maps of the ERA5 reanalysis. (**a**) Climatology of the months October to January (2010–2019). (**b**) Mean of all days meeting the foehn–like conditions. (**c**) Difference of the left two plots. Values in hPa. The dots mark the two investigated cities, Augsburg (green) and Munich (orange).

The definition of foehn-like days can also be used to form air pressure maps with the ERA5 reanalysis data. For the months of October through January, and a maximum of seven lag days, days are selected that have a wind direction between 125° and 235°, wind speed higher than the annual mean, lower relative humidity, and mean daily temperature higher than the respective month. A total of 43% of all rAAs from October to January meet these criteria for at least one day. Figure 4a–c shows the resulting air pressure maps. In the case of foehn events, there is an intensification of the prevailing air pressure constellation. This results in higher air pressure south of the Alps and lower pressure in northern Europe, which allows the Alps to be overflown; thus, affecting the occurrence of foehn in the Bavarian foothills of the Alps.

## 4. Discussion

Foehn is a wind blowing down from mountains, e.g., the Alps, bringing dry and warm air, and having known health-related effects on people with high sensitivity to weather changes [21,22].

We analyzed 152 patients (66% men; 33% women) with a ruptured aortic aneurysm from University Hospital of Augsburg and University Hospital of the Technical University Munich over ten years. Gender and age appear to play a role in aortic rupture according to the available data. Our findings are consistent with that of the literature. The same data were published by an Italian group in 1999. We showed that the aortic aneurysm rupture is also associated with the aorta diameter (high risk at about 7 cm) across all aortic aneurysm morphologies as well as with hypertension. Overall, 55.9% of study patients had high blood pressure in their medical history, which is a known risk factor for dilatation of the aorta wall. Many studies have shown that systolic and diastolic blood pressures were higher in the winter than in the summer, which may increase rAAA incidence rates in the winter [23]. We do not have the proper data to analyze this ourselves. We did not find specific information about the rupture of other aortic morphologies like TAA and TAAA in association with the weather and season in the literature. In our opinion, the number of these aortic ruptures is too small to obtain enough data from a single-center. It is likely that a multicentric study is needed to analyze this. We are the first to show some data on this topic.

The cause for the high mortality of patients with rAA in the winter is, in our opinion, less directly associated with the foehn weather. In the summer, the mortality rate in our investigation is also high, approximately 30%.

The seasonal distribution of pooled rAAs from two universities in southern Germany shows higher incidences of rAAs in summer (28.9%) and autumn (26.3%). The meta-analysis of rAAA in 2019 from Choong et al. identified seasonal association between the rupture of abdominal aortic aneurysms in autumn (incidence rate 26.6%) and winter (incidence rate 26.2%) from 1946 to 2017. We observed the same findings in Augsburg but not in Munich. The seasonal and monthly frequency of rAAs in both cities was highly variable despite the proximity of the two cities (approx. 50 km). In Augsburg, more cases occur in the winter (31.6%) than in the summer (26.6%). Whereas in Munich, rAAs are more common in the summer (31.5%) compared to winter (16.4%). The literature mentions that rAAs occur more frequently at low air pressure and cold air temperatures [24,25,26,27]. In our study, a lower air pressure plays a significant role only in the summer months. This could explain the local differences of rupture frequencies in Augsburg and Munich due to the weather conditions.

Due to the comparison of the occurrence frequencies of weather types in both cities between days of rAAs and days without rAAs during the winter months, we identified at both locations weather types with southerly flow tendency and lower air pressure in the case of rAAs, which correlate with the foehn. More foehn-like events occur in the winter months than in summer. We observed, in January and February, 58% of all rAAs occurred following a foehn-type weather event. In May, over 83% of rAAs were associated with a foehn event. Generally, we observed interannual variability of foehn events during ten years.

The meteorological results showed that the influence of the environmental parameters could be essential for people at risk for the rupture of an aortic aneurysm, depending on their residence. Also, the risk of rAA due to weather phenomena may vary considerably from region to region and from season to season. With climate change likely to increase heat waves and lead to a higher persistence of specific weather types, there could be more of an influence on aortic rupture in the future. The pathophysiological changes that lead to the rupture of an aneurysm during specific weather conditions, like foehn, should be investigated in more detail in further studies. Many elements could invalidate the study’s assumptions, and further investigations and explanations of the correlation between environmental effects and aortic aneurysm rupture rate are necessary for confirmation.

In the future, artificial intelligence could help to predict the high risk of aortic ruptures due to specific weather types.

## 5. Conclusions

We showed the association between aortic aneurysm rupture and specific weather phenomena, such as foehn, and a high incidence of aortic aneurysm ruptures in summer and autumn in southern Germany. The local meteorological weather conditions, in addition to other factors, may play a role in aortic aneurysm rupture.

## Data Availability

No new data were created or analyzed in this study. Data sharing is not applicable to this article.

## Supplementary Materials

The following supporting information can be downloaded at: https://www.mdpi.com/article/10.3390/jcm14093104/s1. Figure S1. Distribution of the patient age and gender with aortic aneurysm rupture from January 2010 to December 2019 at the University Hospital of Augsburg and University Hospital of the Technical University Munich; Figure S2: Comorbidities of patients with ruptured aortic aneurysm; Figure S3: Distribution of different morphologies of rAA in men and women; Figure S4: Different morphologies of ruptured aortic aneurysm and maximum diameter of aorta by rupture; Figure S5: Absolute number of aortic aneurysm ruptures at the University Hospital of Augsburg and University Hospital of the Technical University Munich from January 2010 to December 2019; Figure S6: Monthly distribution of different morphologies of ruptured aortic aneurysm; Figure S7: Mortality from patients with aortic aneurysm rupture in winter (42.6%) and summer (29.8%); Figure S8: Monthly mortality rate (%) of patients with the aortic aneurysm rupture at the University Hospital of Augsburg and University Hospital of the Technical University Munich, Germany.
