# Supplementary figures and images for: Influence of Foehn on Aortic Aneurysm Ruptures in Southern Germany
Source: J Clin Med. 2025 Apr 30;14(9):3104. doi: 10.3390/jcm14093104 (PMC12072723; doi:10.3390/jcm14093104)

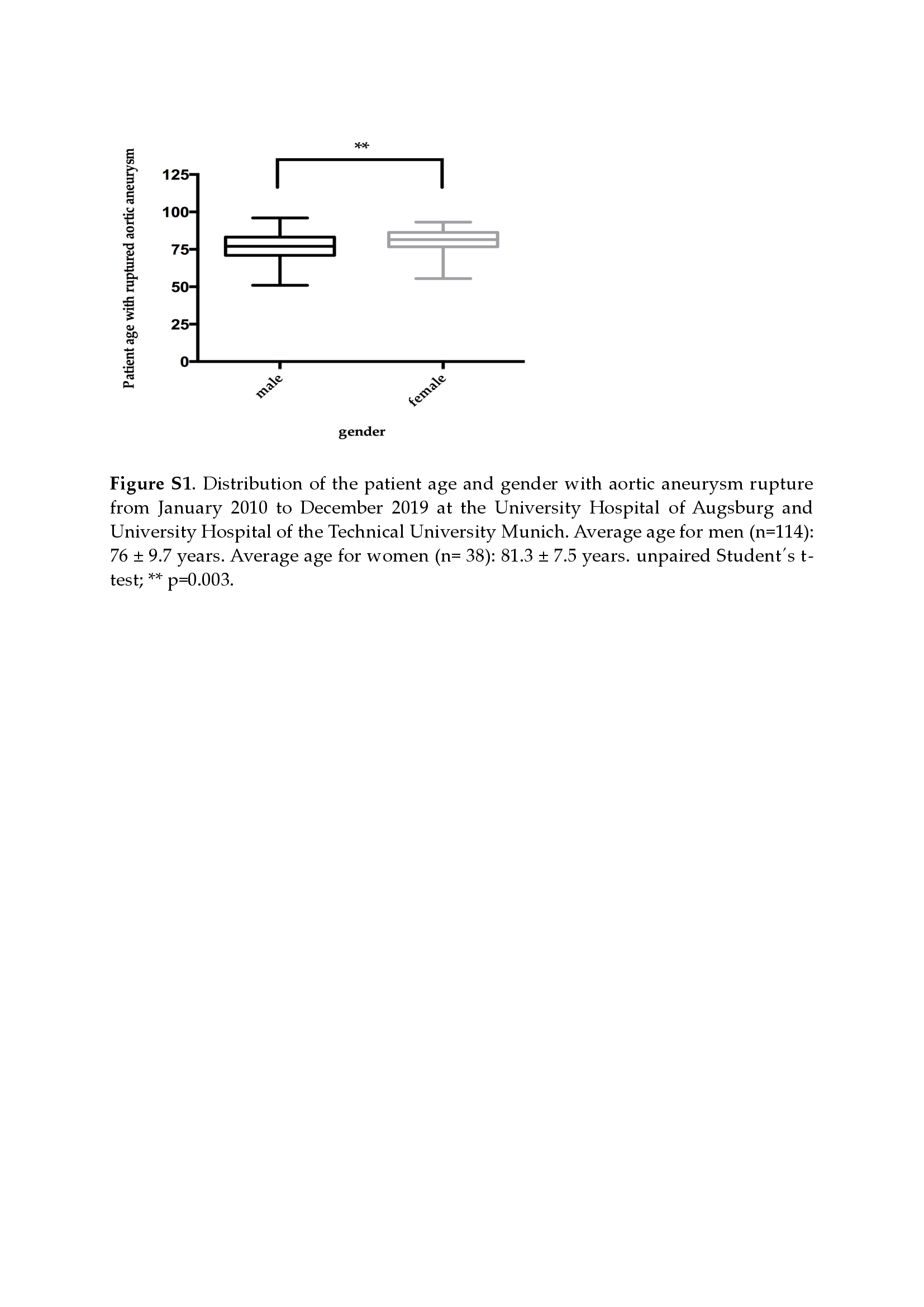

Supplement: Supplementary file 1 [file jcm-14-03104-s001.zip › Figure S1.tiff]

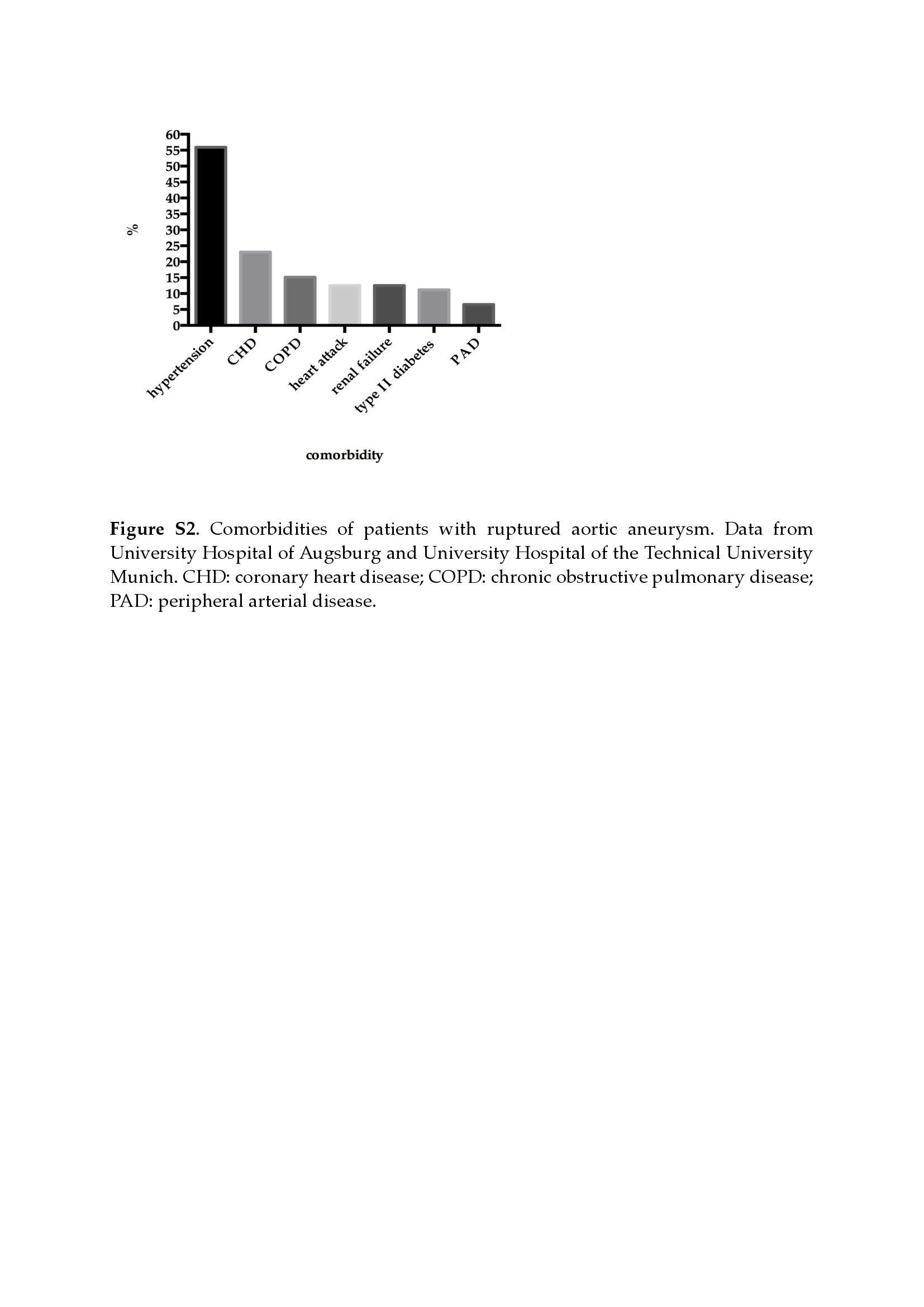

Supplement: Supplementary file 1 [file jcm-14-03104-s001.zip › Figure S2.tiff]

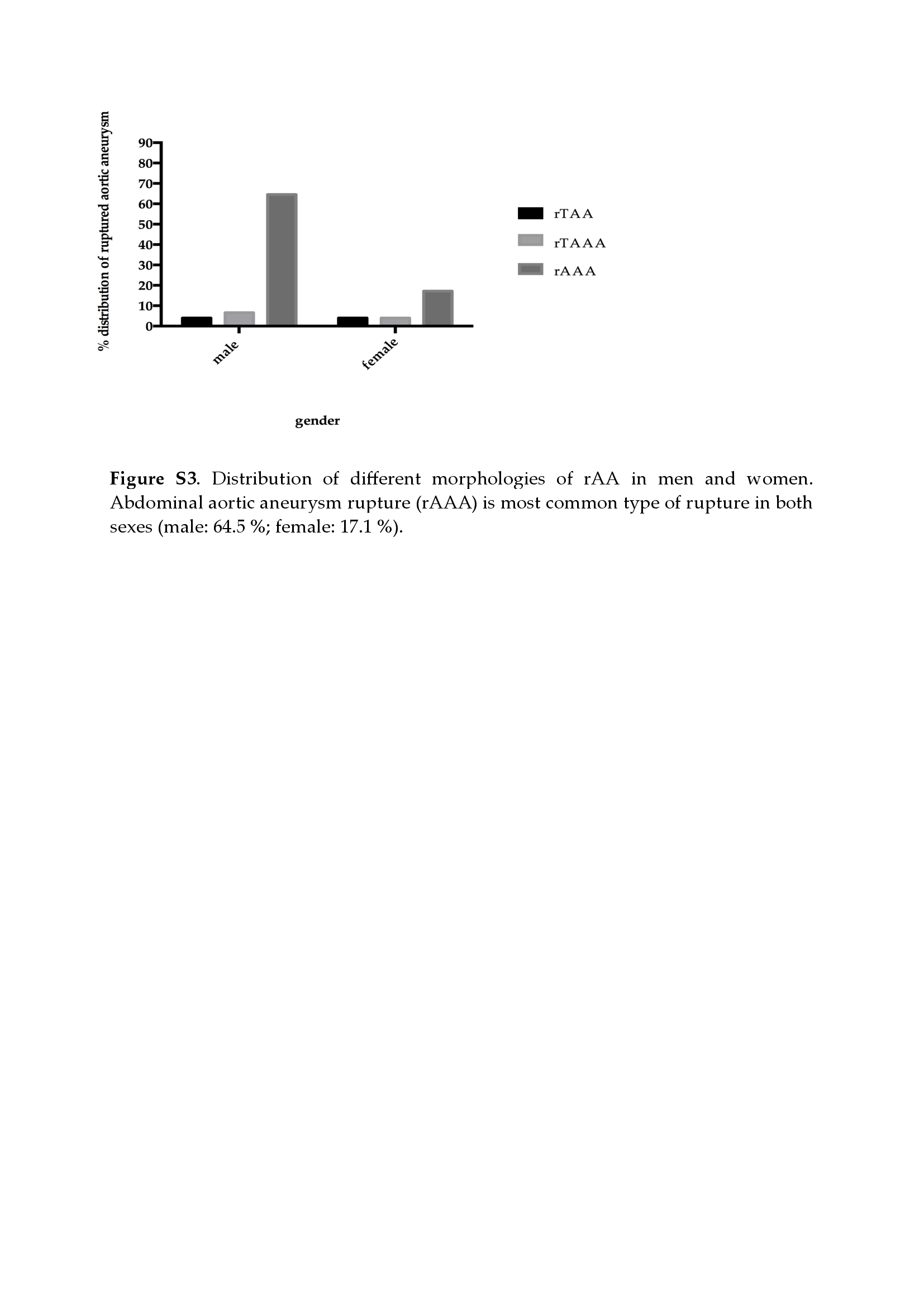

Supplement: Supplementary file 1 [file jcm-14-03104-s001.zip › Figure S3.tiff]

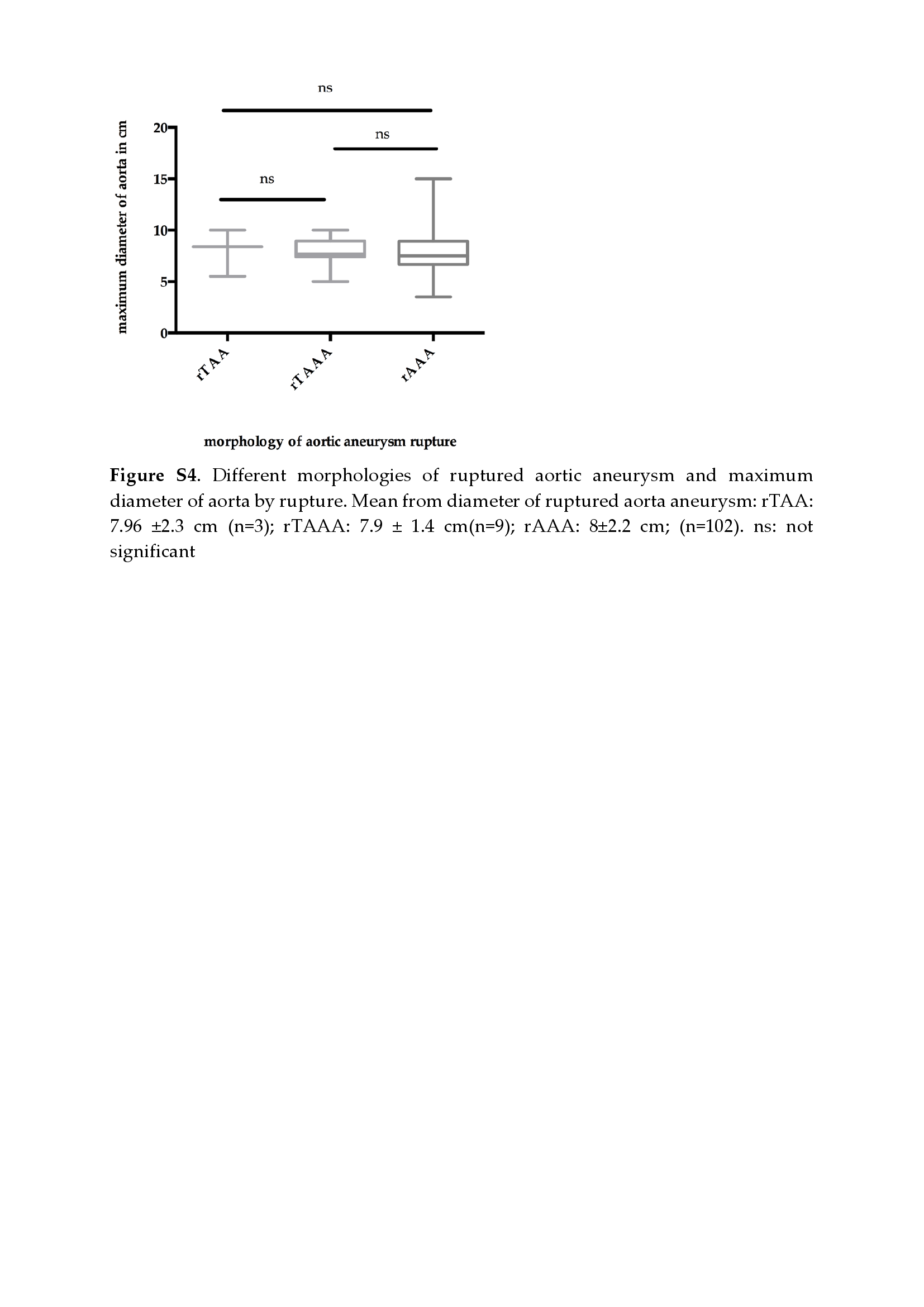

Supplement: Supplementary file 1 [file jcm-14-03104-s001.zip › Figure S4.tiff]

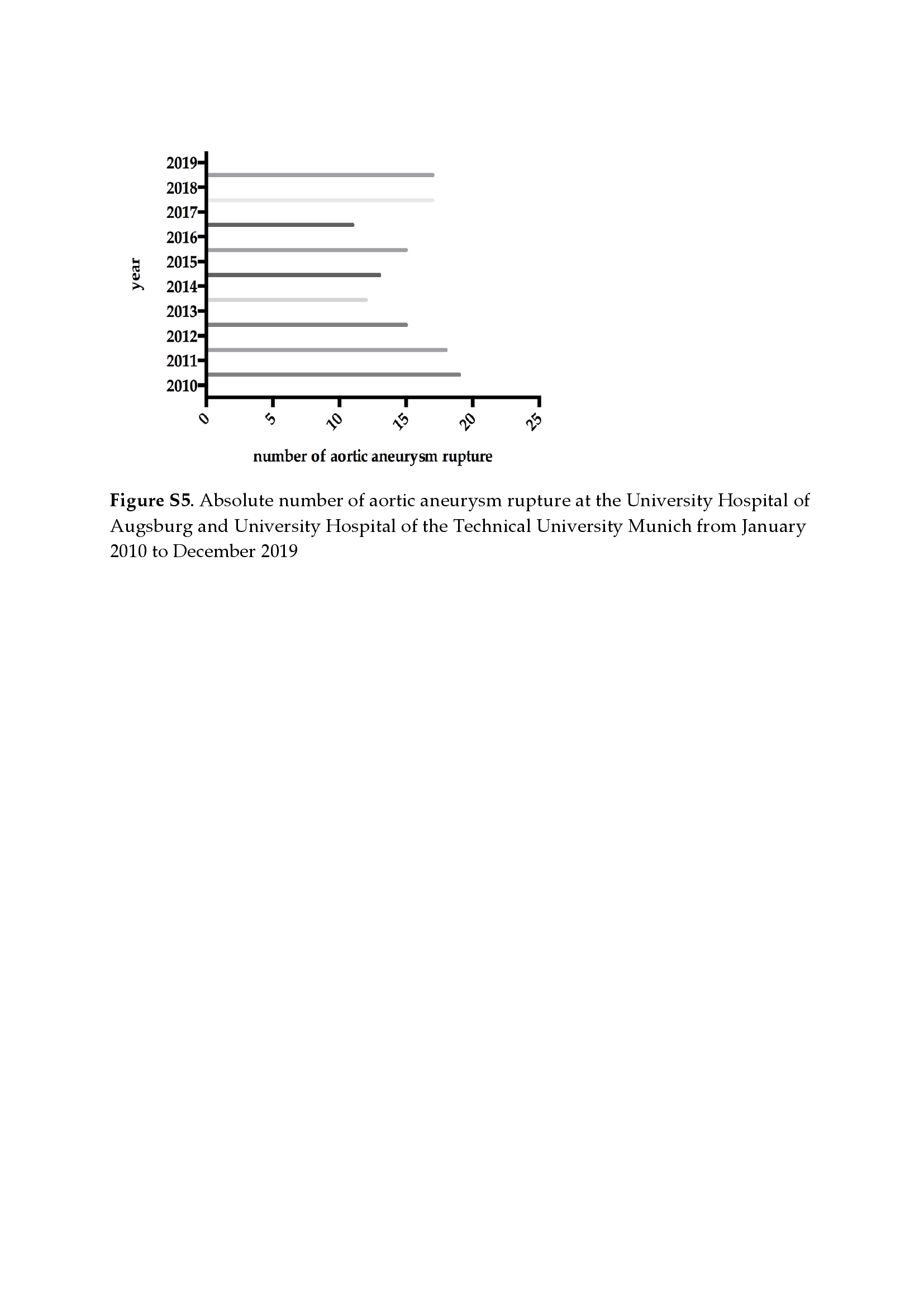

Supplement: Supplementary file 1 [file jcm-14-03104-s001.zip › Figure S5.tiff]

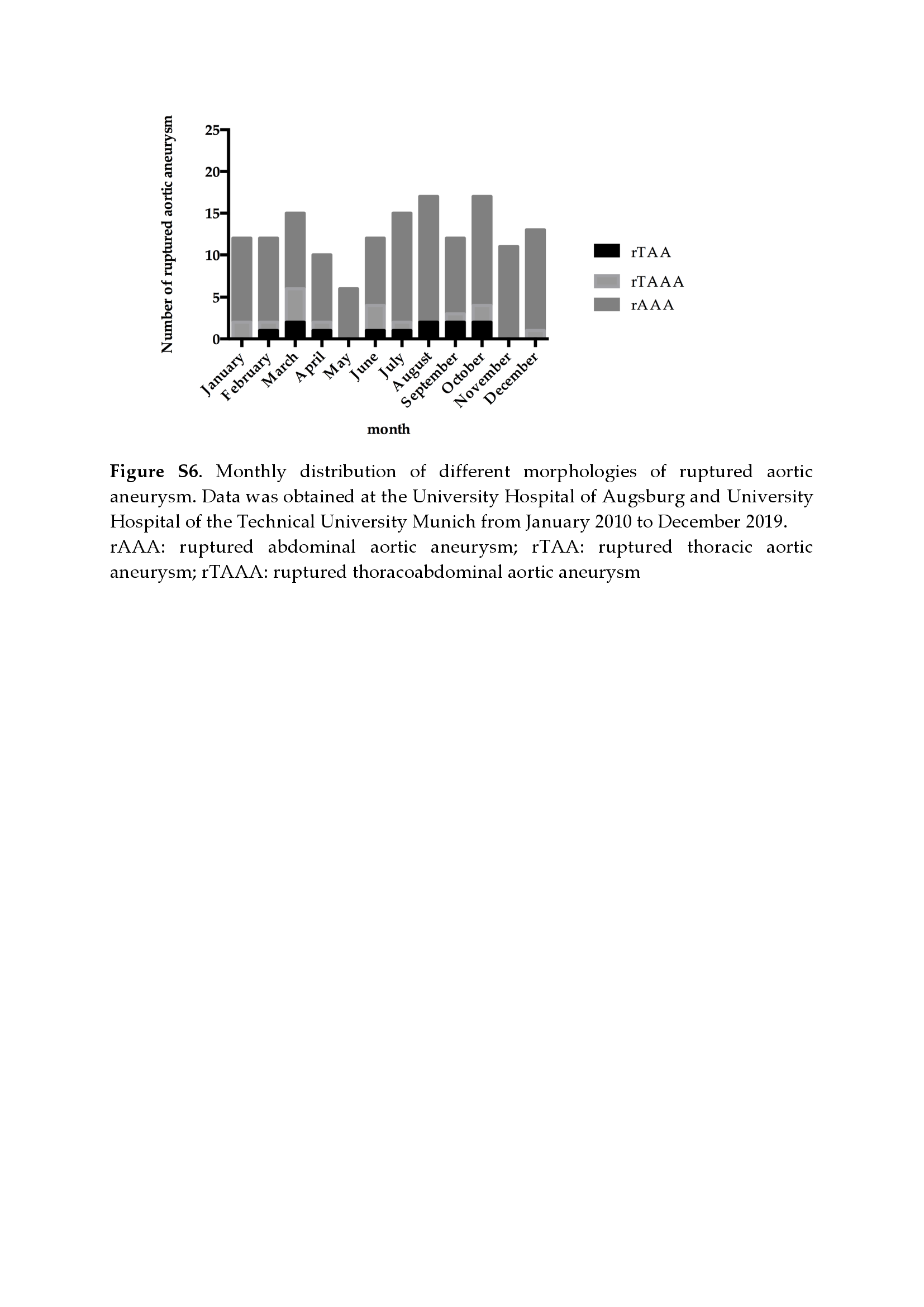

Supplement: Supplementary file 1 [file jcm-14-03104-s001.zip › Figure S6.tiff]

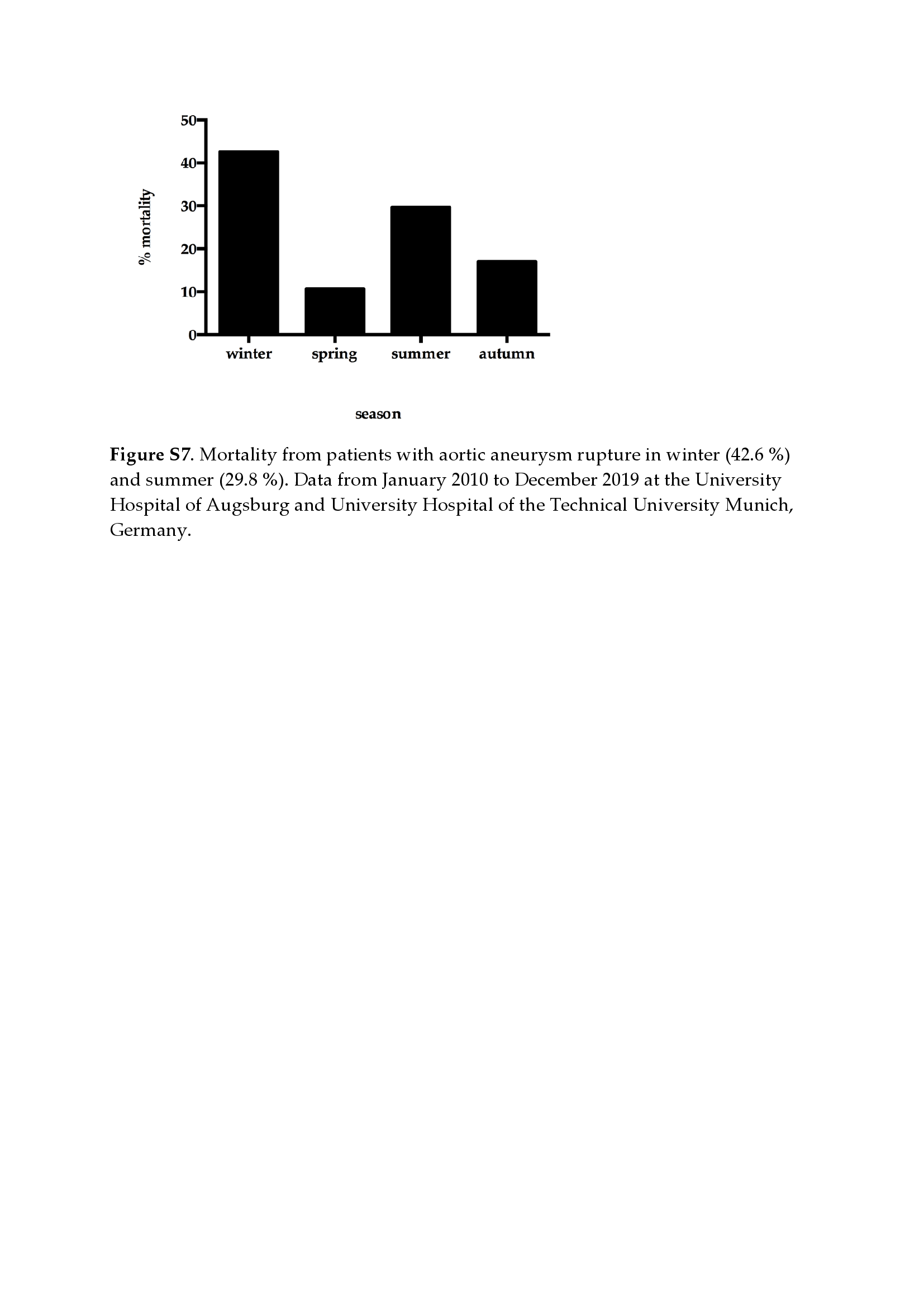

Supplement: Supplementary file 1 [file jcm-14-03104-s001.zip › Figure S7.tiff]

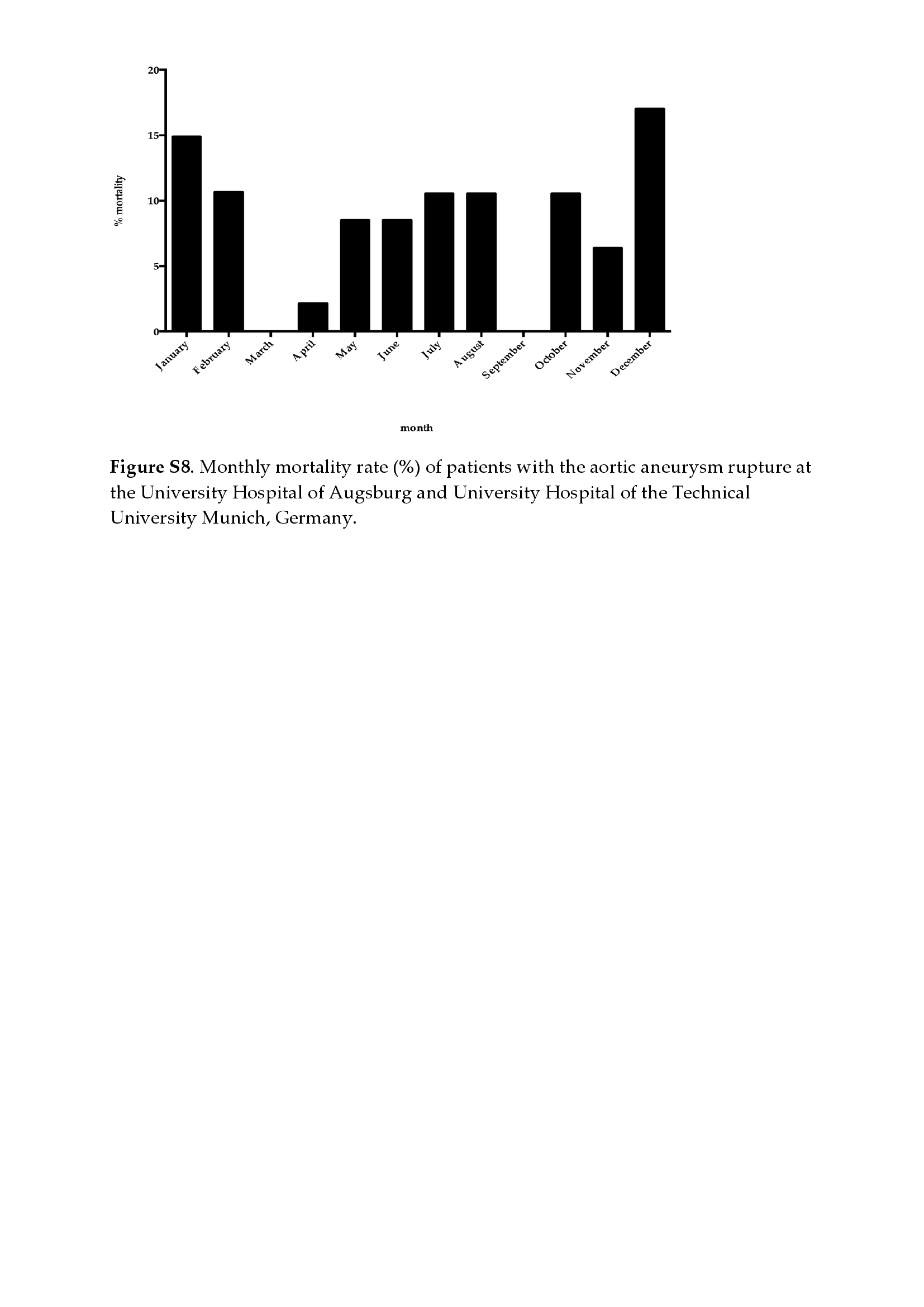

Supplement: Supplementary file 1 [file jcm-14-03104-s001.zip › Figure S8.tiff]
